# The role of neurotensin as a novel biomarker in the endoscopic screening of high-risk population for developing colorectal neoplasia

**DOI:** 10.1007/s13304-017-0464-6

**Published:** 2017-05-30

**Authors:** Christos Kontovounisios, Shengyang Qiu, Shahnawaz Rasheed, Ara Darzi, Paris Tekkis

**Affiliations:** 1 Department of Colorectal Surgery, The Royal Marsden Hospital, Chelsea, London, UK; 2 Department of Surgery and Cancer, Imperial College London, Chelsea and Westminster Hospital Campus, London, UK

**Keywords:** Colorectal cancer, Diagnostic biomarker, Neurotensin

## Abstract

Colorectal cancer screening programs aim at early detection of cancer to reduce incidence rates and mortality. The objective of this study is to identify the role of neurotensin in the endoscopic screening of high-risk population for developing colorectal neoplasia. Blood samples from patients referred for urgent colonoscopy to investigate symptoms suspicious of colorectal cancer were collected. Blood neurotensin levels were measured using enzyme-linked immunosorbent assay. Colonoscopy findings were used as reference for determining the diagnostic accuracy of blood neurotensin. The study comprised 26 patients in total: 12 healthy and 14 with colon pathology (13 high-grade dysplasia adenomatous polyps, 1 adenocarcinoma). There were no statistically significant differences in the clinical and biochemical parameters between colon pathology and healthy group except neurotensin levels. Pathology in colon was associated with 3.7-fold increase in NT levels. In multivariate analysis, patients with pathology in colon have increased serum neurotensin levels compared to controls adjusted for age, gender, BMI and co-morbidities. The value of 12.93 pg/ml is associated with 87.5% sensitivity and 91.7% specificity for discriminating the colon pathology from normal colonic epithelium (*p* = 0.001). Neurotensin plasma values differentiate healthy people from patients suffering from colonic pathologies such as adenomatous polyps and cancer. The use of neurotensin as a potential endoscopic screening tool for identifying high-risk population for developing colorectal cancer is promising, but much has to be done before it is validated in larger scale prospective studies.

## Introduction

Colorectal cancer (CRC) is a worldwide health problem that ranks third in incidence and fourth in mortality with an estimated 1.2 million cases and 0.6 million deaths annually [1, 2]. Mortality can be lowered by both early diagnosis and cancer prevention where the objective of screening is to detect cancer at an early curable stage. The European Union (EU) recommendations for CRC screening is based on tests with quality assurance for the diagnosis and management of patients with screen-detected lesions. In countries with a serious burden of CRC, screening promotes cancer control, provided the services are of high quality. Conventional screening tools include Digital Rectal Examination, Faecal Occult Blood Test (FOBT), Sigmoidoscopy, Colonoscopy, Virtual Colonoscopy and Double Contrast Barium Enema (DCBE). Studies have shown that patients over the age of 60 having had a normal colonoscopy can be followed with FOBT or computerized tomography (CT) pneumocolon every 5 years. This screening process can provide overall the same survival benefit with less morbidity and cost [3]. Colonoscopic surveillance is considered the gold standard in detecting colonic malignancy compared to FOBT; however, low participation rate has questioned its benefit [4].

The last decades have been marked by the accumulation of knowledge about the inner workings of the normal and cancer cell. The discovery and validation of new targets are the foundation and the source of new screening modalities; therefore, efforts have been made to identify blood-based markers for the early detection of CRC. The availability of blood-based, non-invasive tests promises to improve screening compliance and to reduce the morbidity and mortality associated with this malignancy.

Neurotensin (NT) is a 13-amino-acid peptide originally isolated from extracts of bovine hypothalami in 1973 by Carraway and Leeman [5]. The biological effects of NT are known to be through three receptors, two G protein-coupled receptors, neurotensin receptor 1 (NTSR1) and receptor 2 (NTSR2), and a single transmembrane domain sorting receptor 3 (NTSR3) [6, 7]. NT has shown to exert numerous oncogenic effects involved in tumour growth and metastatic spread. These effects are mostly mediated by NTSR1, making the NT/NTSR1 complex an actor in cancer progression. Colorectal cancers are known to possess NT receptors and some produce also NT peptide. Yoshinaga et al. have demonstrated that administration of NT significantly increases mean tumour size, weight and deoxyribonucleic acid (DNA), ribonucleic acid (RNA) and protein contents of the murine colon cancer cell line MC26 [8]. Other studies have demonstrated that the use of NT receptor antagonist have inhibitory effect on human colon cancer cell (LoVo) growth [9] and in cells xenografted into nude mice [10]. In human CRC, NTSR1 was expressed in increasing amounts in adenomas and adenocarcinomas, respectively [11].

Therefore, NT/NTSR1 expression may be involved in the early stages of colonic carcinogenesis and thus could potentially be an additional non-invasive screening tool [12]. At the same time, its non-invasiveness advantage may lead to high-population compliance at a lower cost than the current screening system and with the capacity for early detection of CRC and precursor adenomatous polyps.

This pilot study aimed to investigate whether patients with CRC have a specific pattern of serum NT values compared with healthy controls and determine the feasibility of this factor being used as an additional diagnostic and screening tool for colorectal pathology.

## Materials and methods

The study was conducted in 2015 at Royal Marsden Hospital (RMH) and Chelsea and Westminster Hospital (C&W) and was approved by the NHS Research Ethics Committee. Patients referred for colonoscopic screening/diagnostic colonoscopy at the endoscopy departments of RMH and C/W were prospectively enrolled following receipt of written informed consent, in adherence with local ethics guidelines.

Inclusion criteria were: (1) patients aged 18 years or older, (2) patients referred for colonoscopy under the National Health Service (NHS) Urgent Suspected Colorectal Cancer pathway, (3) patients able to give informed consent. Exclusion criteria were: (1) patients who were pregnant, lactating, or undergoing fertility treatment, (2) patients with known active inflammation or infection or an autoimmune disorder, including inflammatory bowel diseases, (3) patients with known malignancy, (4) patients receiving immunosuppressive treatment or chemotherapy.

For each patient, 5 ml of blood was collected from a peripheral vein 15–30 min prior to participants undergoing colonoscopy. The whole blood was separated into plasma using EDTA as an anticoagulant. Samples were assigned a random sample ID and then centrifuged for 15 min at 1000 relative centrifugal force at 2–8 °C within 30 min of collection. The plasma was collected and immediately stored in a −80 °C freezer.

A competitive enzyme immunoassay (ELISA) kit was used to detect neuropeptide Y peptide according to the manufacturer’s instructions (CUSABIO). This assay utilizes the quantitative sandwich enzyme immunoassay technique. Antibody specific for NT has been pre-coated on to a microplate. Standards and samples were pipetted into the wells and any NT present was bound by the immobilized antibody. After removing any unbound substances, a biotin-conjugated antibody specific for NT was added to the wells. After washing, avidin-conjugated horseradish peroxidase (HRP) was added to the wells. Following a wash to remove any unbound avidin–enzyme reagent, a substrate solution was added to the wells and colour developed in proportion to the amount of NT bound in the initial step. The colour development was stopped and the intensity of the colour was measured using an ELISA microplate reader. The optical density (OD) absorbance was set at 450 nm within 30 min of adding the stop solution. A standard curve was plotted for OD vs. the respective concentration of the standard solutions. The NT concentration (pg/ml) of each sample was extrapolated from the standard curve.

To minimize bias, the colonoscopy findings from each subject were only reviewed after the completion of the ELISA. The colonoscopy results were reviewed by a member of the team (C.K.). The primary outcome of interest was the presence of colorectal polyps, cancers and other significant pathologies detected by colonoscopy. The NT concentration from each blinded sample is matched to the colonoscopy result to allow statistical analysis.

### Statistical analysis

Statistically significant values were considered if *p* < 0.05. Data were presented as mean ± standard error of the mean for quantitative variables and as *n* (%) for qualitative. For quantitative variables, Chi-squared with Fisher’s exact test was used. Test for normal assumption was performed with Shapiro–Wilk test for quantitative variables. Following the normal assumption, it was tested with Student’s *t* test and if this was not the case we used non-parametric Mann–Whitney *U* test. For multivariate analysis, linear regression was used with dependent variable being the level of NT. A receiver operating characteristic (ROC) was used to summarize the performance of two classes among the range of possible variables to establish which value of neurotensin can be used as discriminator between two groups with sufficient sensitivity and specificity.

The Statistical Package for the Social Sciences (SPSS) version 20 was used for data analysis.

## Results

**Fig. 1:**
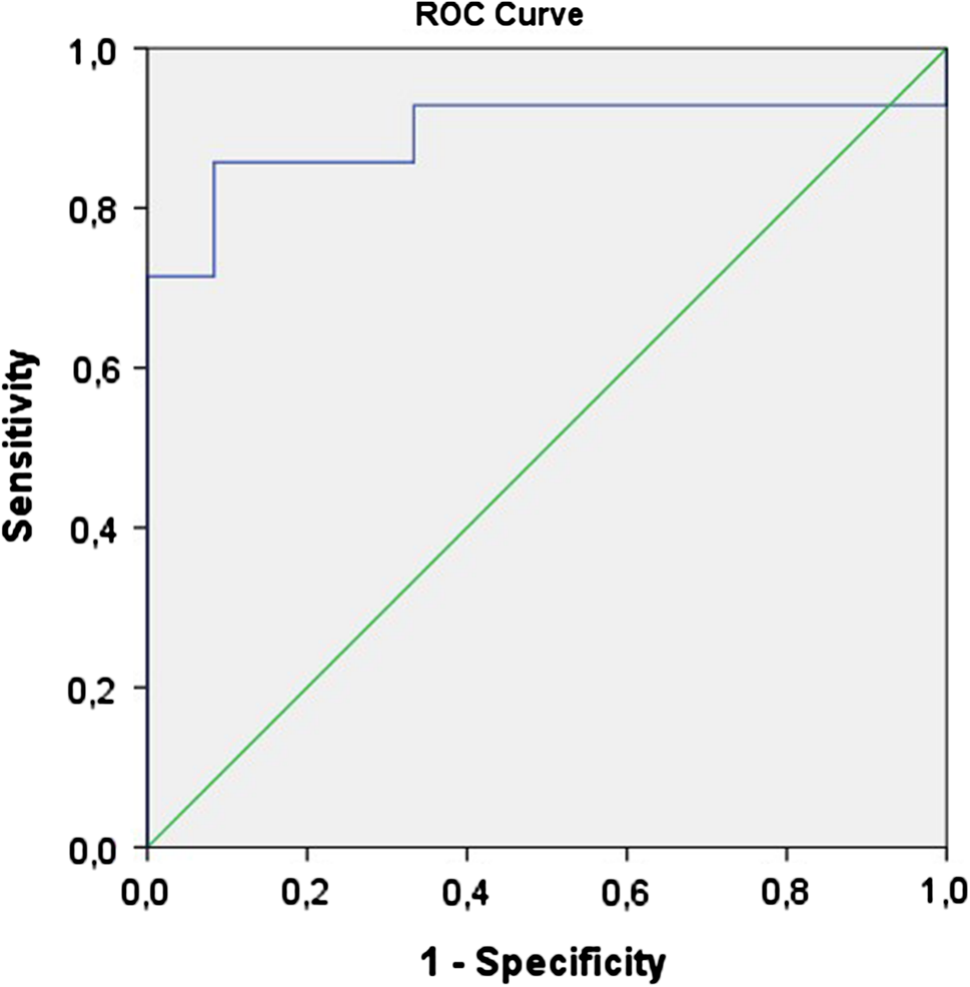
Received operator characteristics (ROC) curve for the sensitivity and specificity of plasma neurotensin level in identifying colorectal polyps and cancer

The study population comprised 26 patients in total: 12 control/healthy subjects with no abnormality or dysplasia on colonoscopy and 14 with colon pathology (13 with high-grade dysplasia adenomatous polyps, 1 with adenocarcinoma). In descriptive analysis, the mean age in years was 55.42, the mean BMI 24, 87 and mean NT level 24 pg/ml. There were no statistically significant differences in the clinical and biochemical parameters between colon pathology group and healthy group. The only significant difference was in neurotensin levels. Pathology in colon was associated with 3.7-fold increase in NT levels (Table 1). In multivariate analysis, patients with pathology in colon have increased serum neurotensin levels compared to controls adjusted for age, gender, BMI and co-morbidities (Table 2). The value of 12.93 pg/ml is associated with 87.5% specificity and 91.7% specificity for discriminating the colon pathology from normal colonic epithelium. The ROC curve was found to be statistically significant (AUC = 0.893, 95% CI 0.749–1, *p* = 0.001) (Fig. 1).

**Table 1:** Characteristics and average blood neurotensin levels of patients who had normal colonoscopy vs. patients with colon pathologies (polyps and cancer)

	Group				*p* value
	Normal		Colon pathology		
	*N*	*N* %	*N*	*N* %	
Gender					
Female	9	75.0	7	50.0	n.s^+^
Male	3	25.0	7	50.0	
Co-morbidities					
No	9	75.0	9	64.3	n.s^+^
Yes	3	25.0	5	35.7	
Smoker					
No	11	91.7	12	85.7	
Yes	1	8.3	2	14.3	n.s^+^
Alcohol consumption					
No	12	100.0	11	78.6	
Yes	0	0.0	3	21.4	n.s^+^

	Mean	SEM	Mean	SEM	*p* value
Age (years)	53.92	5.46	26.71	3.78	n.s*
BMI (kg/m^2^)	22.23	1.41	26.26	1.00	n.s*
Neurotensin (pg/ml)	9	1	37	6	<0.001*

*BMI* body mass index, *SEM* standard error of mean, *pg/ml* picograms per millilitre

^+^Chi-square/Fisher’s exact test, * Mann–Whitney *U* test

**Table 2:** Multivariate regression analysis of effect of the presence of colorectal pathology and other patient factors on increased serum NT levels

	OR	95% CI	*p* value
Group			
Colorectal pathology vs. normal	26.17	12.47 to 39.271	0.001
Age (years)	0.341	−0.108 to 0.79	n.s
Gender			
Female vs. male	5.742	−8.8 to 20.28	n.s
BMI (kg/m^2^)	−0.454	−2.2 to 1.2	n.s
Co-morbidities			
Yes vs. no	6.128	−9.27 to 21.53	n.s

*OR* odds ratio, *95% CI* 95% confidence intervals, *BMI* body mass index

## Discussion and conclusion

Currently, 136,830 new cases of colorectal cancer (CRC) and 50,310 deaths due to CRC have been estimated in the United States in 2014. In the European Union (EU), colorectal cancer is the third most common cancer and the second leading cause of death, accounting for more than 345,000 new cases and 150,000 deaths in 2012. Survivorship is highly dependent at the time of diagnosis since early tumour stage has better therapeutic outcomes. Therefore, early detection of CRC can improve the 5-year survival rate from about 10% up to 90% [13].

The gold standard screening test, colonoscopy, is considered to be invasive and the rate of non-compliance among population is 40% or higher [14]. The faecal occult blood test and faecal immune test are currently the approved non-invasive screening methods, with 60–80% sensitivity and 85–95% specificity [15]. Other blood markers such as carcinoembryonic antigen (CEA) and cancer antigen (CA) 19-9 have lower sensitivity and/or specificity [16]. These markers are predominantly used in the surveillance of colorectal cancer patients but their low screening sensitivity and specificity has excluded from the screening recommendations. Promising results have been published for genes deriving from peripheral blood mononuclear cells (PBMCs) including DNA methylation of specific genes, but more extensive experiments are required to validate their results [17, 18]. Therefore, the combination of the absence of symptoms in early stages of disease, the invasive nature of the gold standard colonoscopy, and the low specificity and sensitivity of faecal blood tests results in late diagnosis of disease with severe burden on patient’s management and in National Health system (NHS) costs.

The ideal marker should have the following qualities:

- High sensitivity and specificity.
- Safe and non-invasive so that it can be broadly accepted by patients.
- Cost effective so that it can be accepted by NHS.
- Easy to measure.
- Should be detected among genders and ethnic groups [19–21].

With the limited suitability of CEA and CA19-9, several candidate proteins have been published as CRC diagnostic markers. A single protein marker, TIMP-1, has detected CRC with 42–65% sensitivity and 95% specificity [22]. Babel et al. reported 43 proteins that could distinguish between CRC patients and healthy controls [23].

The role of NT in CRC is proven in the laboratory setting. Poinot-Chazel et al. has published a mechanism by which NT promotes cell growth in Chinese Hamster Ovary (CHO) cells transformed with human NTRS1 and in colon cancer HT29 cells. He suggested that growth stimulation by NT could involve the coupling of NT receptor to Krox-24 induction via the MAP kinase cascade. This hypothesis is also favoured since Krox-24 is an early response gene producing a zinc-finger transcription factor that targets several genes involved in cell division [24].

Maoret et al. found that administration of NT can stimulate growth in five different human cancer lines (SW480, SW620, HT29, HCT116 and C1.19A) that express NTSR1, but has no effect on cells with absent NTSR1. In SW480 cell lines, NT increased colony formation by approximately 50% [10].

Gui et al. [11] examined NTSR1 mRNA by in situ hybridization in normal colonic mucosa, adenomas and colonic adenocarcinomas. NTSR1 mRNA expression was found to be non-detectable in epithelial cells of normal colonic epithelium but adenomas and adenocarcinomas demonstrated moderate to strong expression (*p* < 0.05). There was higher level of expression in adenocarcinomas compared to adenomas (*p* < 0.05). The intensity of NTSR1 expression was higher in cases where tumour was present in muscularis propria or even gone beyond that layer. On the other hand, expression was less in cases where tumour was localised in the mucosa and submucosa.

Souaze et al. validated the Wnt/APC signalling pathway on the NT1 receptor promoter activation and showed that NT1 receptor gene activation was related to nuclear or cytoplasmic beta-catenin localization. NT1 receptor was absent when beta-catenin was identified in early adenomas of patients with familial adenomatous polyposis, hereditary non-polyposis colorectal cancer and loss of heterozygosity tumours [25].

Sgourakis et al. demonstrated the use of serum NT and interleukin 8 (IL-8) values as a screening tool for colorectal cancer. Fifty-six patients and 15 healthy controls were divided to seven groups according to their disease presentation. Neurotensin (*p* = 0.004) and IL-8 (*p* = 0.029) differed between healthy and colorectal cancer patients. Neurotensin values differentiate the control group from all other groups. The value of plasma NT ≤54.47 pg/ml at presentation selected by receiver ROC curves demonstrated a sensitivity of 77%, specificity of 90%, and an estimate of area under ROC curve of 85% in diagnosing colorectal cancer. This finding is similar to those reported here. Furthermore, it suggests IL-8 should be used complementary to neurotensin due to its lower specificity [26].

In this pilot study, the majority in the colon pathology group were high-grade dysplasia adenomatous polyps, 13/14 patients, which was associated with 3.7-fold increase in NT levels. The value of 12.93 pg/ml is associated with 87.5% sensitivity and 91.7% specificity for discriminating the colon pathology from normal colonic epithelium. To our knowledge, this is one of the first studies to measure plasma neurotensin in humans with the aim of providing a screening system for colorectal cancer by bridging basic research with clinical practice. Our values of neurotensin in adenomatous polyps are lower compared to higher values of other studies which measured NT in adenocarcinoma.

The sample size of this study is small and there is no doubt larger scale studies are required to validate the findings reported here. An inclusion of larger number of CRC patients will also answer the question of whether blood NT level can be used to differentiate cases of colorectal polyps and cancers. In this study, the analysis of the NTSR1 receptor was not performed. These measurements are also pending along with prospective validation of our results which has not yet been applied. Only a single measurement per patient was calculated, but we plan to perform a second measurement in the future. Further to diagnosis and screening, blood tumour markers may also be relevant in the prognosis of CRC [27, 28].

Neurotensin plasma values appeared to differentiate healthy people from patients suffering from colonic pathologies such as adenomatous polyps and cancer, but much has to be done before it is validated in larger scale prospective studies.
